# A Study on the Psychological Impact on Key Caregivers of Traumatic Brain Injury Versus Non-traumatic Brain Injury in Critically Ill Trauma Patients

**DOI:** 10.7759/cureus.71113

**Published:** 2024-10-08

**Authors:** Manjaree Mishra, Shubham K Gupta, Shashi P Mishra, Ravi Shankar Prasad, Mona Srivastava, Arun K Dubey

**Affiliations:** 1 Anaesthesiology, Institute of Medical Sciences, Banaras Hindu University, Varanasi, IND; 2 General Surgery, Institute of Medical Sciences, Banaras Hindu University, Varanasi, IND; 3 Neurological Surgery, Institute of Medical Sciences, Banaras Hindu University, Varanasi, IND; 4 Psychiatry, Institute of Medical Sciences, Banaras Hindu University, Varanasi, IND; 5 Medical Sociology, Institute of Medical Sciences, Banaras Hindu University, Varanasi, IND

**Keywords:** acute stress illness, intensive care unit, non traumatic brain injury, post-traumatic stress disorder, traumatic brain injury

## Abstract

Background: Caregivers of patients admitted in intensive care units may experience acute emotional and psychological stress burden that can manifest as acute psychiatric symptoms and can negatively impact interpersonal relationships and work performance. The aim of this study is to elucidate the socioeconomic burden of trauma and the effect of psychological stress on key caregivers between traumatic brain injury (TBI) and non-TBI patients.

Methods: The study was conducted on 200 caregivers of critically ill trauma patients admitted to the trauma ICU for at least 48 hours and were divided into two groups: Group 1- Patients with TBI with Trauma ICU admission of more than 48 hours and Group 2- Non-TBI patients (chest trauma, abdominal trauma, etc.) with Trauma ICU admission of more than 48 hours. The key caregivers in two groups were subjected to Critical Care Family Need Inventory (CCFNI) and to National Stressful Events Survey Acute Stress Disorder Short Scale (NSESSS) to measure the needs and severity of acute stress symptoms respectively) at 48 hours of ICU admission, day seven, day 15, and day 30 (follow-up). The statistical analysis was performed using SPSSv23 (IBM Corp., Armonk, NY, USA).

Results: Motor vehicle collision was the most common mode of injury among patients in both groups. The sociodemographic parameters of the caregivers were comparable in both groups. Predominantly the patients were males while key caregivers were females. In Group 1 caregivers experienced higher family burden and severe psychological distress at ICU. At the time of admission, needs of caregivers in all the domains (support, comfort, information, accessibility and reassurance) were comparable in both groups. On day seven, the needs for information, accessibility, and reassurance in the TBI group were significantly higher than in the non-TBI group (p<0.01). On day 15, the needs for support, information, accessibility, and reassurance in the TBI group were significantly higher than in the non-TBI group (p≤0.001). On day 30, the needs for support, information, accessibility, and reassurance in the TBI group were significantly higher than in the non-TBI group (p<0.001) but need for comfort was higher in the non-TBI group (p<0.05). Caregivers of patients with TBI had higher stress burden and higher NSESSS than that of non-TBI (p<0.05).

Conclusion: We concluded that key caregivers of patients with TBI have a high stress burden, highlighting the importance of providing psychological support to this group.

## Introduction

Trauma, being the leading cause of disability and death under 45 years of age, has significant physical, psycho-social and financial burden on not just the patients but also on their caregivers, severely affecting the quality of life. Severity of injury has been directly related to the length of stay, need for ventilator support and critical care, treatment cost, disability and death. Family members of critically injured trauma patients who are admitted to an intensive care unit (ICU) may find the ICU to be a very strange and unsettling place [1]. Because of the unplanned hospitalization, the family has psycho-social and financial concerns including fear of losing their loved ones or their integrity. Urgent decision-making for critically ill and sedated patients has compelled the involvement of families in patient management. These include decisions on end-of-life care, the use of life-sustaining therapies, etc [2]. Besides this even after discharge from the ICU stay, caregivers and patients may have long-term posttraumatic stress-related symptoms [3].

Stress can alter behavior and lead to weariness, poor eating habits, decreased sleep quantity or quality, a worsening of health issues, and peri-traumatic and post-traumatic stress disorder. There are plenty of studies on psychological stress and development of post-traumatic stress disorder (PTSD) in military personnel returning from combat/war. These seeded in the studies on the psychological impact on the primary caregiver in critically sick trauma patients especially in those with traumatic brain injury (TBI). Though TBI is the most common type of trauma requiring ICU admission and has quite longer length of stay, Non-traumatic brain injury (NTBI) contributes as another subgroup for ICU bed occupancy. Long-term effects of trauma, specifically the development of PTSD have been extensively studied. However, development of acute stress symptoms and their severity have not been studied much in key caregivers of trauma patients admitted to the ICU. Moreover, the needs of caregivers of NTBI patients and the severity of acute psychological stress may not be the same as TBI patients and are yet to be quantified [4,5]. There is no work to date to support our hypothesis that measurement of needs of family members as measured by Critical Care Family Need Inventory (CCFNI) and the burden of acute psychological stress using National Stressful Events Survey Acute Stress Disorder Short Scale (NSESSS), are different between caregivers of TBI and NTBI patients [6,7]. The aim of the study is to explore the psychological burden in key caregivers of trauma patients. We hypothesize that the psychological burden is different in key caregivers of TBI vs non TBI patients considering the unexpected and delayed recovery in the case of TBI patients and possibly the family status of the trauma victims. Therefore, we intend to define and compare CCFNI needs and NSESSS amongst the caregivers of TBI and of those without TBI following at least 48 hours of ICU stay.

## Materials and methods

The study was conducted at the Intensive Care Unit, Trauma Centre, Institute of Medical Sciences, Banaras Hindu University, Varanasi. After receiving ethical approval from institute ethical committee (approval No. Dean/2021/EC/2396) and obtaining written informed consent, 200 caregivers of critically ill trauma patients admitted to the trauma ICU for at least 48 hours were enrolled. They were divided into two groups. Group 1 included caregivers of patients with TBI and Group 2 included caregivers of non-TBI patients (chest trauma, abdominal trauma etc.) admitted to Trauma ICU for more than 48 hours. 

Only patients with key caregivers/family members of age 18 years or more were enrolled in the study. The key caregiver is close or blood relative of patient and in charge of financial support and decision making. Caregiver with previous/current history of any psychiatric disorder or antipsychotic medications, caregivers of patients who died within 48 hours of ICU admission were excluded from the study.

Patient parameters like age, gender, mechanism of injury, injury severity score (ISS), length of hospitalization, length of ICU stay were studied. Socio-demographic parameters of caregivers like age, gender, educational, marital and employment status, family income and relationship of caregivers with patient were also recorded.

Finally, the key caregivers in two groups were subjected to CCFNI at 48 hours of ICU admission, at day seven, day 15, and day 30 (follow-up), which was revised by Leske in 1991 [6]. The CCFNI contains 45 needs items, which are divided into five dimensions: support, comfort, information, accessibility, and reassurance. The scoring scale ranged from 0 to 4 and was based on their importance. Higher score indicates higher needs among key caregiver in both the groups [7]. The English version of CCFNI scale was used to assess the needs among key caregiver.

The severity of stress burden on caregivers of both the groups was assessed by NSESSS [8-10]. The NSESSS was administered to all patients who met inclusion criteria, a seven-item score for each item was measured on five-point scale with the total score ranging from 0 to 28. Seven items, each rated on a five-point scale (0=Not at all; 1=A little bit; 2=Moderately; 3=Quite a bit, and 4=Extremely) added to gain a total raw score ranging between 0 to 28, with higher scores indicating greater severity. The prorated score takes into account for number of items actually answered and is obtained by formula: (total raw sum x 7)/number of items actually answered. Here, we have used prorated score for assessment. Higher score indicates the severity of acute stress among key caregiver in both groups (Table 1).

**Table 1:** National Stressful Events Survey Acute Stress Disorder Short Scale (NSESSS)

S.No.	How much have you been bothered during the PAST SEVEN (7) DAYS by each of the following problems that occurred or became worse after an extremely stressful event/experience?	Each question to be scored 0-5
1	Having “flashbacks,” that is, you suddenly acted or felt as if a stressful experience from the past was happening all over again (for example, you reexperienced parts of a stressful experience by seeing, hearing, smelling, or physically feeling parts of the experience)?	0: Not at all, 1: A little bit, 2: Moderately, 3: Quite a bit, 4: Extremely
2	Feeling very emotionally upset when something reminded you of a stressful experience?	
3	Feeling detached or distant from yourself, your body, your physical surroundings, or your memories?	
4	Trying to avoid thoughts, feelings, or physical sensations that reminded you of a stressful experience?	
5	Being "super alert,” on guard, or constantly on the lookout for danger?	
6	Feeling jumpy or easily startled when you hear an unexpected noise?	
7	Being extremely irritable or angry to the point where you yelled at other people, got into fights, or destroyed things?	

The statistical analysis was performed using SPSS Version 23.0. (IBM Corp., Armonk, NY, USA). Simple descriptive statistics was used (mean ± standard deviation for quantitative variables, and frequency with percentage distribution for categorized variables). The statistical analysis was carried out for various categorical parameters using chi square test and Fischer’s Exact Test. For comparing means of two groups Student’s t-test and Mann Whitney test were performed. For paired samples, Paired Student’s t test was used. P-value <0.05 is considered as statistically significant.

## Results

The mean ages of patients versus caregivers were 38.2 years (SD 11.8) versus 42.38 years (SD 12.44) in the TBI group and 34.6 years (SD 10.4) versus 43.51 years (SD 13.63) in the non-TBI group. While the patients mostly were males, key caregivers were usually females. Mean ISS was significantly higher in the TBI group i.e. 24 versus 18.4 in the non-TBI group (p<0.05). Motor vehicle collision was the most common mechanism of injury in both groups. Mean length of ICU stay and hospital stay was significantly higher in the TBI group as compared to the non-TBI group (p<0.01) (Table 2).

**Table 2:** Demographic characteristics of patients in both groups ISS: injury severity score, TBI: traumatic brain injury

Variable	Group A: TBI patients	Group B: Non-TBI patients
Mean Age (SD)	38.2 (11.8)	34.6 (10.4)
Male	74	78
Mean ISS	24.04 (6.24)	18.4 (4.56)
Mechanism of injury		
Motor vehicle collision	63	69
Fall from height	18	14
Physical assault	16	15
Firearm injury	3	2
Mean length of ICU stay (SD)	16.54 (11.61)	7.23 (4.22)
Mean length of Hospital stay	28.8 (18.43)	12.4 (8.74)

The socio-demographic characteristics of the caregivers were comparable among both groups (Table 3). Majority of caregivers of patients were female in both groups and were usually the parents of the patients or a spouse. Two-thirds of the key caregivers were earning members of the family. There was no statistically significant difference noted between the two groups as far as demographics of caregivers are concerned.

**Table 3:** Demographic characteristics of caregivers in both groups TBI: traumatic brain injury

Variable	Caregivers of patients with TBI with Trauma ICU stay of >48 hrs (n=100)	Caregivers of non-TBI patients with Trauma ICU stay of >48 hrs (n=100)
Age (years)	42.38±12.44	43.51±13.63
Gender		
Male	42	39
Female	58	61
Education		
Illiterate	21	19
High school	36	35
10+2 or Degree	43	46
Marital status		
Married	72	71
Unmarried	22	25
Others	6	4
Occupation		
Earning	66	62
Dependent	34	38
Family Income		
Low	31	34
Middle	33	36
High	36	30
Domicile		
Urban	57	61
Rural	43	39
Caregiver relationship		
Mother	31	29
Father	18	19
Sister	15	17
Brother	13	13
Wife	10	9
Daughter	3	3
Husband	7	8
Son	3	2

Table 4 shows comparison of needs of family caregivers of group 1 and 2 patients using the CCFNI within 48 hours of ICU admission, day seven, day 15 and day 30. At the time of admission, needs of caregivers in all the domains (support, comfort, information, accessibility and reassurance) were comparable in both groups. On day seven, the needs for information, accessibility, and reassurance in the TBI group were significantly higher than in the non-TBI group (p<0.01). On day 15, the needs for support, information, accessibility, and reassurance in the TBI group were significantly higher than in the non-TBI group (p≤0.001). On day 30, the needs for support, information, accessibility, and reassurance in the TBI group were significantly higher than in the non-TBI group (p<0.001) but needs for comfort was higher in the non-TBI group (p<0.05).

**Table 4:** Scores of five Critical Care Family Need Inventory (CCFNI) needs dimensions in caregivers of patients with TBI and caregivers of non-TBI patients with trauma TBI: traumatic brain injury

	Caregivers of patients with TBI with Trauma ICU stay of >48 hrs (n=100)	Caregivers of non-TBI patients with Trauma ICU stay of >48 hrs (n=100)	p-value
Within 48 hours of ICU admission			
Support	2.11±0.23	2.12±0.32	0.799
Comfort	2.21±0.53	2.23±0.48	0.788
Information	3.36±0.38	3.40±0.41	0.475
Accessibility	2.13±0.28	2.16±0.31	0.473
Reassurance	3.21±0.41	3.10±0.42	0.062
Day 7			
Support	2.17±0.43	2.13±0.52	0.554
Comfort	2.36±0.61	2.38±0.56	0.809
Information	3.40±0.41	2.51±0.44	<0.001
Accessibility	2.32±0.35	2.18±0.32	0.003
Reassurance	4.08±0.47	3.11±0.42	<0.001
Day 15			
Support	2.36±0.47	2.14±0.47	0.001
Comfort	2.38±0.54	2.52±0.52	0.063
Information	3.53±0.45	2.63±0.41	<0.001
Accessibility	2.74±0.33	2.31±0.31	<0.001
Reassurance	4.11±0.41	3.11±0.48	<0.001
Day 30			
Support	2.49±0.46	2.14±0.52	<0.001
Comfort	2.36±0.61	2.54±0.62	0.039
Information	3.62±0.46	2.70±0.43	<0.001
Accessibility	2.90±0.31	2.29±0.37	<0.001
Reassurance	4.21±0.47	3.08±0.52	<0.001

Caregivers of patients with TBI had higher stress burden and higher NSESSS than that of non-TBI (p<0.05). While there was no statistically significant difference noted in NSESSS score of caregivers of TBI patients at day three following ICU admission, they tend to fall higher on NSESSS scale at days seven, 15 and 30 as compared to their non-TBI counterparts (p<0.001) (Table 5).

**Table 5:** National Stressful Events Survey Acute Stress Disorder Short Scale (NSESSS) for DSM-5 in caregivers of patients with TBI and caregivers of non-TBI patients with trauma TBI: traumatic brain injury, DSM-5: Diagnostic and Statistical Manual of Mental Disorders, Fifth Edition

	Caregivers of patients with TBI with Trauma ICU stay of >48 hrs (n=100)	Caregivers of non-TBI patients with Trauma ICU stay of >48 hrs (n=100)	p-value
Within 48 hours of ICU admission	19.5±5.3	18.5±4.3	0.246
Day 7	16.5±8.3	10.8±5.5	<0.001
Day 15	11.0±7.5	7.2±5.3	<0.001
Day 30	9.8±6.25	4.5±5.5	<0.001

## Discussion

In the context of TBI, psychosocial and mental health concerns of caregivers are little explored in India, with a focus on critical care in particular. Most of the studies addressing the psychological burden were conducted on ICU admitted patients of medical etiology, only a few studied both medical as well as surgical patients. These surgical patients too had non-traumatic etiology. Our study is distinctive and attempts to through some light on the psychological impact on caregivers of ICU patients admitted following trauma. To the best of our knowledge, there is no study comparing needs (CCFNI) and acute stress (NSESSS) of caregivers of TBI patients with those of non-TBI patients in the Indian population.

The findings of the current study indicated that female caregivers had a greater share of the burden of care than male caregivers. In providing day-to-day care for ICU-admitted patients, parents or spouses were commonly involved. Warren et al. also found female predominance and parents or spouses as the most common caregivers. In coherence with our study, they too found no statistical differences in socio-demographic profiles between the two groups [11].

The study found a strong correlation between the burden of family caregiving in the areas of finances, disrupted family activities, family leisure time, and family interaction patterns, which was more amongst those caring for TBI patients as compared to non-TBI. The differences between the groups were not much and statistically insignificant initially at day three (i.e. after 48 hrs of ICU admission). However, as the weeks passed by, the differences in needs particularly in the domains of information and reassurance increased significantly, with greater needs of caregivers of TBI patients (Table 3). It can be implied that the caregivers' physical and mental well-being has been significantly impacted because of the disease and its course. Additionally, while providing care in the ICU, the caregiver went through significant psychological suffering.

According to earlier research, as families come to terms with the reality of the injury soon after a TBI, they frequently exhibit a range of mourning behaviors. Denial of the injury's effects on the injured family member is a common first-world problem for many families. Depression, rage, and anxiety can later accompany grief [12]. On enquiring about the potential non-medial causes of acute stress, we found that the majority of people are responsible for paying the medical bills (investigation, bed charges, medications, etc), therefore the caregivers frequently also have to deal with financial crises. Being simultaneously involved in medico-legal issues, key caregivers specifically the male ones had to go through the stress of police inquest, paperwork and constant feelings of insecurity and fear about what will happen next. Their chance of developing acute stress and numerous other psychological problems in the long run is significantly increased by these triggers.

We discovered that family caregivers who were under a lot of psychological strain had bad interpersonal interactions and bad doctor-patient and nurse-patient relationships [13]. Conflicts hampered care, which had a negative effect on patients' ability to heal. Several instances involved family members arguing about medical expense issues or disagreeing on how to provide care. Some family caregivers may not have chosen the best course of therapy because they were unable to adequately appreciate the severity of the patient's condition. Therefore, it's critical to comprehend the psychological conditions and traits that affect the psychological issues experienced by family caregivers. With additional knowledge, more focused strategies may be developed to assist family caregivers in maintaining a positive outlook, taking care of their daily requirements, and enhancing the outcome for these patients.

We found that information, accessibility, and reassurance were the three most crucial demands for family caregivers. More precisely, they required a sense of hope [14], knowledge of the anticipated result, assurance that the patient was receiving the finest care [15], and at least once a day access to the most recent patient information. Families wanted the doctors to be honest with them about the patient's prognosis, but they also wanted doctors to offer them reason for optimism. Support and comfort, in contrast, were the least significant need dimensions for family caregivers.

Families paid great heed to words of encouragement and relied on skilled assistance to inspire them to hold onto their hope. Most frequently (almost 50% of the time), support was noted as coming from nurses [13]. To address the needs of the family for support, comfort, information, accessibility, and assurance, nurses are responsible for offering or coordinating interventions, sharing information, organizing family conferences, controlling visiting hours, and bedside family/patient contact. As a result of spending so many intimate hours with patients and their families, nurses may be highly regarded. The two most important characteristics of nursing care were competence and effective communication. Therefore, it is important to stress these skills to nurses so they can treat TBI patients holistically.

## Conclusions

We came to the conclusion that there is a high stress burden among caregivers of TBI patients, which may result in PTSD, underscoring the significance of offering psychological support to this group. Assessment of caregivers' family stress and psychological discomfort at the ICU is urgently needed, as is prompt psychosocial support. Future research should concentrate on ways to lower psychological problems, increase family trust in the medical system, and eventually enhance patient outcomes. Also the findings of this study highlight the need for regular medical dialogues between ICU physicians and caregivers as well as stringent counselling services on a regular basis in order to alleviate the psychological stress in caregivers.

The limitations of this study are that the study is single institution based and non-randomized with short-term follow-up. The authors feel that a multi-institutional well randomized study with longer follow-up may give better insight into the issue of concern.
